# The Effectiveness of Cognitive Training Using Electroencephalography in Acute Stroke Cases

**DOI:** 10.3390/jcm15114376

**Published:** 2026-06-05

**Authors:** Yi-Hsuan Wu, Chiung-Fang Chang, Wei-Hsien Chien

**Affiliations:** 1 11G, Far Eastern Memorial Hospital, New Taipei City 220, Taiwan; femh92042@femh.org.tw; 2 MS Program in Transdisciplinary Long Term Care, College of Medicine, Fu Jen Catholic University, New Taipei City 242062, Taiwan; 3 Department of Medical Research, Far Eastern Memorial Hospital, New Taipei City 220216, Taiwan; changcf@femh.org.tw; 4 Department of Occupational Therapy, College of Medicine, Fu Jen Catholic University, New Taipei City 242062, Taiwan

**Keywords:** stroke, electroencephalogram, Mini-Mental State Examination, Conners’ Continuous Performance Test

## Abstract

**Background/Objectives**: Approximately 17 million individuals worldwide experience stroke annually. Stroke-induced cerebral hypoxia or infarction can impair multiple cognitive domains. This study aims to monitor the cognitive abilities of patients with acute stroke through the intervention of electroencephalogram (EEG) devices. **Methods**: Patients from the neurology ward were invited to participate after obtaining study approval from the research ethics committees of a medical center in northern Taiwan. Participation was explained to the eligible individuals, and only those who met the criteria and signed the informed consent form were included. The participants were those who agreed to undergo 10 sessions of the EEG training. Ultimately, 30 valid samples were collected. The effectiveness of the intervention was analyzed using the pre- and post-test results of the Mini-Mental State Examination (MMSE) and Conners’ Continuous Performance Test (CPT3). **Results**: After 10 EEG intervention sessions, the patients showed significant differences in the pre- and post-test results of the MMSE and CPT3 (*p* = 0.0001 and *p* = 0.007, respectively). The EEG training suggests a possible association with changes in cognitive performance following stroke. **Conclusions**: EEG-based interventions may be potentially associated with cognitive improvement. The effects appeared similar across patient subgroups; however, given the pilot nature of this study and the absence of a control group, the findings should be interpreted cautiously. Further well-designed controlled studies are needed to confirm these preliminary observations and evaluate their clinical applicability.

## 1. Introduction

Stroke is one of the leading causes of mortality and disability worldwide. The World Health Organization (WHO) reports that approximately 17 million people suffer from stroke annually, with over 6 million deaths or long-term disabilities [1]. These statistics highlight stroke as a major global public health concern. Among the various complications associated with stroke, cognitive impairment is one of the most prevalent yet often underrecognized outcomes. Epidemiological data indicate that 12–18% of patients with mild cognitive impairment (MCI) progress to dementia within one year [2,3]. In addition, those with concomitant Alzheimer’s disease pathology exhibit a substantially elevated risk of vascular cognitive impairment [4]. Therefore, it underscores the importance of early detection and intervention in MCI, which may slow disease progression and improve patient quality of life.

Stroke can be broadly categorized into three major types: ischemic stroke, hemorrhagic stroke, and transient ischemic attack (TIA). Ischemic stroke, accounting for nearly 80% of cases, includes thrombotic and embolic subtypes that are closely associated with atherosclerosis and cardiac disorders such as atrial fibrillation [5,6,7]. Thrombotic stroke arises from progressive arterial narrowing and plaque rupture leading to vessel occlusion [5], while embolic stroke results from clots originating from distant sites, most often the heart. Individuals with atrial fibrillation or valvular disease are particularly prone to embolic events due to irregular blood flow and increased thrombus formation [6,7]. Hemorrhagic stroke, on the other hand, arises from the rupture of cerebral blood vessels, resulting in intracranial bleeding and is often associated with higher mortality rates [8,9]. Lastly, transient ischemic attack (TIA) is characterized by brief episodes of cerebral ischemia caused by temporary vascular occlusion. Although symptoms usually resolve within 24 h, TIAs are critical warning signs for future stroke events.

The clinical course of stroke is typically divided into three phases—hyperacute (within 24 h), acute (within 7 days), and subacute or post-acute (within 3 months)—with diagnosis based on a detailed medical history, neurological examination, and neuroimaging such as computed tomography (CT) or magnetic resonance imaging (MRI) to rapidly determine the etiology and guide treatment. Acute management strategies include thrombolysis within three hours of onset, antiplatelet or anticoagulant therapy, and surgical interventions in cases of hemorrhage [10,11]. Despite advances in acute care, a substantial proportion of survivors continue to experience long-term deficit, particularly in cognition and motor function, highlighting the urgent need for effective rehabilitation strategies. Previous studies have suggested that early rehabilitation during the acute and subacute stages may facilitate neuroplasticity and support functional recovery after stroke [12,13]. Therefore, early-stage rehabilitation interventions may have potential relevance in promoting recovery among patients with stroke.

Conventional rehabilitation encompasses physical, occupational, and speech therapy, aiming to improve functional outcomes and quality of life [13]. In recent years, brain–EEG-based training technologies have emerged as promising adjunctive tools in neurorehabilitation [14,15]. Studies have demonstrated that EEG, integrated with virtual reality and functional electrical stimulation, can facilitate neural plasticity and improve both motor and cognitive functions [16,17]. Noninvasive electroencephalography (EEG)-based devices, such as the BrainLink system, have been applied in attention monitoring, memory training, and cognitive enhancement, with evidence supporting improvements in attention, working memory, and executive function [18,19,20]. Recent reviews have further highlighted the potential of integrated neurofeedback and biofeedback approaches in improving cognitive, behavioral, and psychological outcomes across various conditions, with EEG-based methods being the most widely used due to their low cost, non-invasiveness, and ease of use. These approaches encompass various protocols targeting different aspects of brain activity; however, further studies with greater methodological rigor are needed to confirm their effectiveness [21,22]. Moreover, EEG-based neurofeedback has been shown to enhance cognitive recovery and motor rehabilitation in stroke survivors [20,23]. The EEG device can interpret psychological activities that people cannot express verbally [24]. Through using the EEG devices, researchers can obtain valuable information about the brain’s functions and operations.

In addition, neurocognitive assessment tools such as the Conners Continuous Performance Test, Third Edition (CPT3), have been employed to differentiate MCI from subjective cognitive decline (SCD). Evidence suggests that patients with MCI display significantly prolonged reaction times and impaired vigilance, indicating that sustained attention metrics may serve as early biomarkers for cognitive decline [16]. Given the relatively high prevalence of MCI in the geriatric population, developing health strategies is essential—not only for accurate diagnosis and effective treatment but also for improving awareness and management of its consequences [25,26]. Taken together, stroke-related cognitive impairment represents a significant clinical and public health challenge. Post-stroke cognitive impairment has also been associated with increased risks of long-term dependency, depression, institutionalization, and mortality [27]. Although emerging evidence highlights the potential of EEG-based interventions in rehabilitation, relatively fewer studies have evaluated consumer-grade EEG neurofeedback systems for cognitive recovery in stroke populations within real-world clinical settings [28]. Consumer-grade EEG devices generally refer to portable, low-cost, and user-friendly EEG systems designed for non-clinical or home-based applications. Compared with research-grade EEG systems, these devices typically utilize fewer electrodes, simplified electrode placement, and proprietary signal-processing algorithms, making them more accessible and feasible for real-world neurofeedback and rehabilitation applications. Recent study has also reported that BCI- and EEG-based rehabilitation may improve motor function and cognitive network activity after stroke through neuroplastic mechanisms [14]. Therefore, the present study aims to investigate the effects of EEG interventions on cognitive function in stroke patients and to evaluate their clinical feasibility and therapeutic potential.

Previous study has suggested that factors such as age, sex, baseline cognitive status, and stroke characteristics may influence rehabilitation outcomes after stroke [29]. Therefore, subgroup analyses based on age, sex, and clinical diagnosis were further explored to examine whether EEG-based intervention demonstrated differential effects across patient characteristics. In addition, although EEG-based neurofeedback interventions have been increasingly investigated, prior studies remain heterogeneous with respect to patient populations, intervention protocols, and outcome measures. Many previous studies have primarily focused on chronic-stage patients or controlled experimental settings [14,28,30]. In contrast, the present study specifically evaluated acute stroke patients in a real-world clinical inpatient setting. Furthermore, objective neurocognitive assessments, including MMSE and CPT3, were incorporated to evaluate both global cognitive function and attention performance. This approach provides preliminary clinical insights into the feasibility and potential applicability of EEG-based cognitive training during the acute phase of stroke recovery.

## 2. Materials and Methods

### 2.1. Subject

The study subjects were acute stroke cases admitted to the neurology ward of a medical center in New Taipei City. All subjects were patients with acute to subacute stroke and were enrolled within 3 months after stroke onset. The study was approved by the Institutional Review Board (IRB: 112177-E) and employed a purposive sampling method. Inclusion criteria were as follows: (1) a single acute stroke episode; (2) Mini-Mental State Examination (MMSE) scores between 18 and 23; (3) ability to communicate; and (4) ability to maintain a seated position for at least 30 min. Exclusion criteria included moderate to severe dementia, speech impairments, or aggressive behaviors. Patients with recurrent stroke episodes were excluded from the study.

Baseline demographic and clinical characteristics, including age, sex and stroke type (cerebrovascular accident [CVA] or intracerebral hemorrhage [ICH]) were collected at baseline. Stroke diagnosis was confirmed by neurologists based on clinical symptoms and neuroimaging findings, including computed tomography (CT) or magnetic resonance imaging (MRI). Participants were categorized into two age groups (<70 and ≥70 years) based on previous studies suggesting that older stroke survivors may have increased vulnerability to cognitive decline and poorer rehabilitation outcomes [29].

### 2.2. Research Instruments and Methods

The BRAINLINK EEG device (Model BL002V2.0; Sheng Hong Precision Technology Co., Ltd., Taichung City, Taiwan) was used to acquire EEG signals during the intervention. The device utilizes a TGAM chip to capture raw EEG signals from a frontal electrode configuration (EEG, GND, REF). EEG signals were processed in real time using the proprietary algorithm embedded in the device, which extracts frequency-domain features, including delta, theta, alpha, and beta band activity. The EEG device analyzes multiple EEG frequency-band components, including delta, theta, low alpha, high alpha, low beta, high beta, low gamma, and high gamma activity. These frequency-band signals are processed using the manufacturer’s proprietary algorithm and converted into composite neurofeedback indices representing attention and relaxation states. The attention index reflects the participant’s relative attentional engagement during task performance, whereas the relaxation index reflects relative relaxation states during neurofeedback training. These indices are continuously updated in real time and displayed as quantitative values ranging from 0 to 100 during the intervention sessions.

Neurofeedback was provided to participants through visual feedback based on these indices, allowing participants to modulate their brain activity during the training sessions. Participants were instructed to maintain attention and engagement according to the feedback provided by the system. Although the detailed algorithm of the proprietary system is not publicly available, previous studies have applied neurofeedback-based training using consumer-grade EEG devices, supporting the feasibility of using such indices for cognitive training [31,32].

EEG signals were obtained using a frontal electrode configuration, with reference and ground electrodes integrated within the device system. Recordings were performed under standardized conditions with participants in a seated position. Signal processing and feature extraction were conducted using the device’s built-in proprietary algorithm. Although the detailed preprocessing procedures, including artifact removal (e.g., ocular and motion artifacts), are not fully disclosed, the system applies a consistent and standardized processing pipeline across all participants. The processed EEG signals were converted into quantitative indices for subsequent statistical analysis.

### 2.3. Mini-Mental State Examination (MMSE)

MMSE, one of the most widely used tools for assessing cognitive function, is frequently utilized in both clinical and research settings, particularly in older adults. It evaluates seven key domains of cognitive ability: orientation (to person, time, and place), attention, memory, calculation and writing abilities, language skills (including fluency, comprehension, and repetition), recall ability, and constructive abilities [33]. The MMSE has a maximum score of 30 points and is commonly used to screen for cognitive impairment. Scores are interpreted as follows: (A) 24–30 points: Intact cognitive function; (B) 18–23 points: MCI; (C) 0–17 points: Severe cognitive impairment.

### 2.4. Conners’ Continuous Performance Test, 3rd Edition (CPT3)

The CPT3 is a computerized assessment tool designed to evaluate attention-related problems in individuals aged 8 years and older [34,35]. It measures performance across four key aspects of attention: inattention, sustained attention, impulsivity, and vigilance. This tool is widely used for the diagnostic evaluation of attention deficit hyperactivity disorder and other attention-related difficulties. Scores range from below 40 to above 70, with different scores indicating varying levels of attention performance. The CPT3 employs visual stimuli for testing. The target stimuli consist of all letters of the alphabet (26 letters) except the letter X. Participants are instructed to press the spacebar as quickly as possible when a target stimulus appears, while refraining from responding to the non-target stimulus (the letter X). Stimulus intervals vary between 1, 2, and 4 s, and the interval changes after every 20 stimuli. The ratio of target to non-target stimuli is 16:4, and the total test is divided into 6 regions. Each region contains 48 target and 12 non-target stimuli, with a total test duration of 14 min. Table 1 summarizes the meaning of CPT3.

### 2.5. Study Procedure

**Figure 1:**
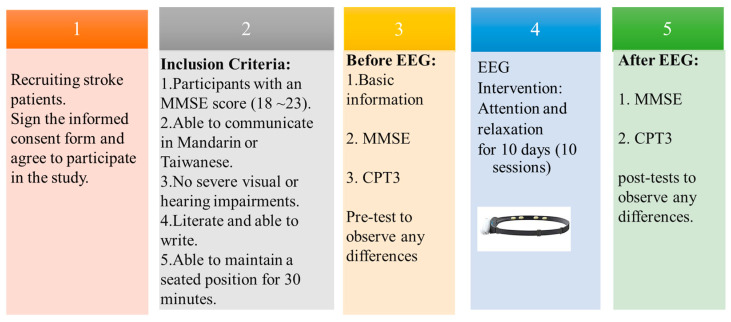
Flow diagram of the study procedure. The process includes five sequential stages: (**1**,**2**) participant recruitment and inclusion criteria, (**3**) baseline information before EEG measurement, (**4**) EEG recording, and (**5**) post-EEG assessment.

We provided all participants with a detailed explanation of the study and obtained their informed consent. The participants then completed the MMSE and CPT3 as pre-tests. Subsequently, they underwent 10 sessions of EEG-based training, with each session lasting 30 min. During hospitalization, participants continued to receive standard medical management and routine rehabilitation programs according to their individual clinical conditions, including pharmacological treatment, physical therapy, and occupational therapy. The EEG-based training program primarily focused on attention and relaxation exercises. No additional intensive cognitive rehabilitation protocol was introduced specifically for this study (Figure 1). The training program focused on attention and relaxation exercises. After completing the training sessions, they underwent post-tests to assess changes in cognitive performance.

Prior to each session, we instructed the participants to ensure that the metal electrode of the EEG device was securely placed against their forehead. The EEG device was paired with an iPad via Bluetooth. Once the connection was established, the participants engaged in two types of tasks designed to assess focus and relaxation abilities. For focus training, we asked the participants to focus on a virtual barrel displayed in the game. The higher their focus level, the faster the barrel would explode. For relaxation training, the participants practiced relaxation by controlling a floating ball. The ball would ascend higher and remain aloft for a longer duration based on their relaxation levels. The system automatically recorded the ball’s height and the time taken to maintain it.

### 2.6. Analytical Plan

Statistical analysis was conducted using SPSS 25 (IBM. Co, Ltd., Chicago, IL, USA). Paired t-tests for dependent samples were used to compare pre- and post-intervention outcomes. The normality of data distribution was assessed prior to analysis. A significance level of 0.05 was adopted. Paired t-tests for dependent samples were used to compare pre- and post-intervention outcomes. The normality of paired differences was assessed using the Shapiro–Wilk test and Q-Q plots prior to analysis. A significance level of 0.05 was adopted. In addition to *p*-values, effect sizes (Cohen’s d) were calculated to evaluate the magnitude of the observed differences.

## 3. Results

### 3.1. EEG Intervention Analysis

The baseline characteristics of the study population are summarized in Table 2. A total of 30 patients were included in this study. The majority of participants had hemorrhagic stroke (73.3%), while 26.7% had ischemic stroke. All participants were recruited within 3 months after stroke onset, corresponding to the subacute phase of recovery. The mean baseline cognitive function, assessed using the MMSE, was 20.83 ± 1.82, indicating mild to moderate cognitive impairment at study entry.

**Figure 2:**
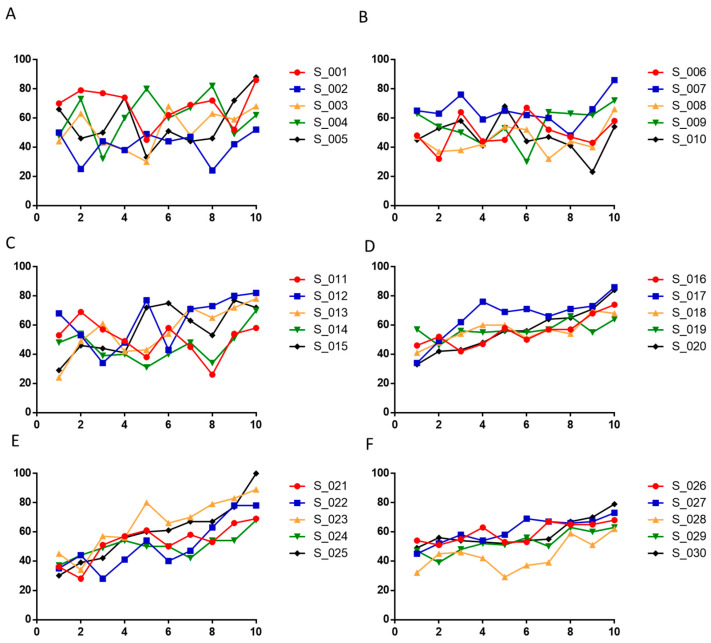
Electroencephalogram (EEG)-derived attention index measurements of 30 participants. The *x*-axis indicates the measurement sessions (10 trials in total), and the *y*-axis denotes the EEG-derived attention scores expressed as percentages (0–100%). Panels (**A**–**F**) display the results for participants grouped in sets of five. Each plot represents five participants, with each line corresponding to an individual subject. The EEG parameter presented in this figure represents the attention index generated by the device algorithm (range 0–100) rather than raw EEG waveforms or spectral EEG features. The attention index was derived from integrated frontal EEG frequency-band components, including delta, theta, alpha, beta, and gamma activities, processed using the manufacturer’s proprietary algorithm. The values shown in this figure represent composite neurofeedback indices related to attentional engagement rather than raw EEG waveforms, spectral power measurements, or single-frequency electrophysiological parameters.

A total of 30 participants completed the EEG assessments. Figure 2 illustrates the EEG measurements across 10 sessions, with the *X*-axis representing the number of sessions (1–10) and the *Y*-axis indicating EEG focus scores (0–100). To avoid over-crowding in the visualization, we divided the participants into groups of five and created separate line charts for each group (Figure 2A–F). Table 3 presents a comparison of the EEG-derived attention index between the first and tenth sessions. The results showed that the mean attention score increased from 46.30 in the first session to 72.57 in the tenth session (*p* < 0.001), suggesting a significant improvement over the course of the intervention sessions.

### 3.2. EEG Intervention and Analysis of Pre- and Post-Test Differences in MMSE Scores and Influencing Factors

**Figure 3:**
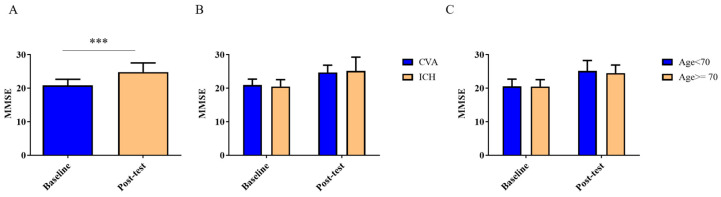
Mini-Mental State Examination (MMSE) scores before EEG intervention (baseline) and after EEG intervention (post-test). (**A**) All participants, (**B**) participants grouped by stroke type (cerebrovascular accident [CVA] or intracerebral hemorrhage [ICH]), and (**C**) participants grouped by age. *** *p* < 0.0001 indicates statistically significant differences.

The mean MMSE pre-test score was 20.83, indicating MCI. As shown in Table 3 and Figure 3A, the MMSE post-test yielded a significantly higher mean score of 24.80. Prior to statistical analysis, the normality of paired differences was assessed using the Shapiro–Wilk test and Q-Q plots, which showed no significant deviation from normality (W = 0.9660, *p* = 0.4359). A paired t-test showed a statistically significant difference between the pre- and post-test scores (*p* < 0.001), suggesting an improvement in MMSE scores following the EEG intervention sessions. A large effect size was observed for the MMSE improvement (Cohen’s d = 1.94). Further correlation analysis between the pre- and post-test MMSE scores revealed a coefficient of determination (R^2^ = 0.67), indicating a strong and statistically significant correlation between the two measures (Table 4). These findings provide preliminary evidence of a potential association between EEG-based intervention and improved cognitive performance among participants with MCI.

In addition, we also categorized clinical diagnoses into two groups: cerebrovascular accident (CVA) and intracerebral hemorrhage (ICH). We conducted a t-test analysis to compare the groups. The MMSE pre-test scores ranged from 20.50 to 20.95 with a *p*-value of 0.555, indicating no significant difference. The MMSE post-test scores ranged from 24.68 to 25.13 with a *p*-value of 0.779, also showing no significant difference. These results suggest that MMSE scores were comparable between participants with CVA and ICH diagnoses, and the observed differences were not influenced by the type of clinical diagnosis (Figure 3B).

Moreover, we categorized the participants into two age groups: under 70 years and 70 years or older. The t-test analysis, conducted to examine differences between these groups, showed no significant difference in the MMSE baseline scores, which ranged from 20.50 to 20.57, with a *p*-value of 0.482. The MMSE post-test scores ranged from 24.50 to 25.14, with a *p*-value of 0.532, also showing no significant difference. These findings suggest that MMSE scores were similar across age groups (Figure 3C).

### 3.3. EEG Intervention and Analysis of Pre- and Post-Test Differences in CPT3 Scores and Influencing Factors

**Figure 4:**
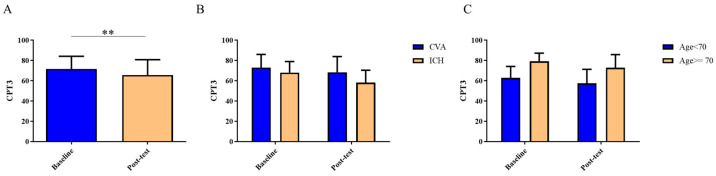
Conners’ Continuous Performance Test 3rd Edition (CPT-3) scores before EEG intervention (baseline) and after EEG intervention (post-test). (**A**) All participants, (**B**) participants grouped by stroke type (cerebrovascular accident [CVA] or intracerebral hemorrhage [ICH]), and (**C**) participants grouped by age. ** *p* < 0.01 indicates statistically significant differences.

The mean CPT3 pre-test score was 71.63, whereas the mean post-test score was 65.63. The Shapiro–Wilk test demonstrated that the CPT score differences were normally dis-tributed (W = 0.9712, *p* = 0.5731), indicating no significant deviation from normality. Therefore, parametric statistical analysis using paired *t*-tests was considered appropriate. A paired *t*-test revealed a statistically significant difference between the pre- and post-test scores (*p* = 0.007), indicating that the EEG intervention may be associated with improvements in CPT3 performance and attention-related cognitive function (Table 5, Figure 4A). Further correlation analysis between the pre- and post-test CPT3 scores revealed a coefficient of determination (R^2^ = 0.69), indicating a strong and statistically significant correlation. These findings provide preliminary evidence of a potential association between EEG intervention and improved attention performance as measured by CPT3 scores.

First, participants were categorized into two groups based on clinical diagnosis: CVA and ICH. We compared the groups with a t-test analysis. CPT3 pre-test scores ranged from 68.00 to 72.95, with a *p*-value of 0.35, indicating no significant difference. CPT3 post-test scores ranged from 58.13 to 68.36, with a *p*-value of 0.106, also showing no significant difference. Changes in CPT3 scores (pre- to post-test) ranged from −9.88 to −4.59, with a *p*-value of 0.265, indicating no significant difference between diagnostic groups (Figure 4B). These results suggest that CPT3 scores were comparable between CVA and ICH groups and were not influenced by differences in clinical diagnosis.

In addition, participants were divided into two age groups: under 70 years and 70 years or older. We compared these groups using a t-test analysis. CPT3 pre-test scores ranged from 62.86 to 79.31, with a *p*-value of 0.000, indicating a significant difference. CPT3 post-test scores ranged from 57.50 to 72.75, with a *p*-value of 0.004, also showing a significant difference. The changes in CPT3 scores (baseline to post-test) ranged from −6.56 to −5.36, with a *p*-value of 0.777, indicating no significant difference in score improvement between the two age groups (Figure 4C).

## 4. Discussion

This study aimed to investigate the potential association of EEG intervention on the rehabilitation of patients with stroke, focusing on analyzing differences. Our study suggests that EEG-based intervention may be associated with improvements in stroke patients across different age groups and diagnostic categories. The results indicated that although differences between subgroups were not statistically significant, all groups demonstrated significant improvements in scores. These findings provide preliminary evidence suggesting that EEG-based intervention may be associated with cognitive improvement in stroke patients across different age groups and diagnostic categories. The absence of significant subgroup differences may partially reflect the limited sample size and insufficient statistical power.

Our study investigated the effects of an EEG combined with memory and attention training games, similar to Lee et al.’s study [18]. In their research, an eight-week intervention consisting of 24 sessions, each lasting 30 min, produced significant improvements in immediate memory, visuospatial/construction abilities, attention, and delayed memory. However, Lee et al. assessed participants using the MMSE and a self-reported questionnaire at the final EEG training session. The participants rated items on a seven-point Likert scale (1 = strongly disagree to 7 = strongly agree), which relied on subjective evaluations. In contrast, we also employed the MMSE but supplemented it with the CPT3 to provide a more objective assessment of cognitive function.

The observed improvements in cognitive function followed 10 EEG intervention sessions in this study. These findings are consistent with previous studies using portable EEG headband systems, which have shown that real-time EEG-derived indices can distinguish between attention and relaxation states, supporting the feasibility of neurofeedback-based approaches in behavioral and cognitive training [36]. However, it has a few limitations. Primarily, our results did not clarify whether increasing the number of intervention sessions could lead to long-term improvements in cognitive performance. To further evaluate differences in the degree of cognitive improvement, future research could benefit from incorporating a healthy control group. Two previous studies have included a healthy control group as part of their experimental design [17,30].

This study has several limitations that should be acknowledged. First, the absence of a control group limits the ability to fully exclude the potential influence of repeated cognitive testing effects, nonspecific treatment effects, or spontaneous recovery after stroke. Although all participants had experienced only a single stroke event and were enrolled during the acute stage after stroke, residual confounding factors may still exist. Therefore, the observed cognitive improvements cannot be attributed solely to EEG-based intervention. In addition, participants continued to receive standard medical management and routine rehabilitation therapies during hospitalization, including physical therapy, occupational therapy, speech therapy, and pharmacological treatment. Therefore, the observed cognitive improvements may not be entirely attributable to EEG-based intervention alone. Nevertheless, routine rehabilitation programs in the acute inpatient setting were generally limited in duration and primarily focused on standard supportive care during hospitalization. In contrast, the EEG-based cognitive training intervention in the present study was implemented in a structured and continuous manner for 10 consecutive days, with each session lasting approximately 30 min. This relatively intensive and standardized intervention protocol may have provided additional opportunities for sustained cognitive engagement and attention-focused neurofeedback training during the acute stroke rehabilitation period. Second, the relatively small sample size may limit the generalizability of the findings. Third, our results did not clarify whether increasing the number of intervention sessions could lead to sustained long-term improvements in cognitive performance. Future research incorporating healthy control, sham, or active comparison groups, as well as longer follow-up periods, is warranted to further validate these findings.

Two previous studies have included a healthy control group as part of their experimental design [18,37]. For example, Lee et al. [18] incorporated a waitlist control group in their randomized pilot study and demonstrated improvements in memory and attention outcomes following EEG-based cognitive training, supporting the feasibility of controlled neurofeedback intervention designs. Similarly, Kober et al. [37] included stroke patients receiving treatment as usual as a control condition and reported that neurofeedback training improved several domains of memory performance in post-stroke patients. These findings support the feasibility and potential clinical utility of controlled neurofeedback intervention designs in cognitive rehabilitation.

This study investigated the potential association of EEG-based cognitive training with cognitive outcomes in patients with acute stroke and demonstrated significant improvements in both global cognitive function (MMSE) and attention performance (CPT3) following 10 training sessions. The post-test MMSE average score reached 24 points, which falls within the normal range. These findings provide preliminary clinical evidence supporting the feasibility of EEG-based neurofeedback as an adjunctive rehabilitation approach in acute stroke settings.

Compared with previous studies, this study contributes by focusing specifically on acute-stage stroke patients in a real-world clinical inpatient environment. Additionally, the integration of MMSE and CPT3 enabled a more comprehensive evaluation of both global cognitive function and attention performance. However, the results should be interpreted cautiously due to the absence of a control group. Our participants simultaneously received physical, occupational, and pharmacological therapies during their hospitalization, which may have influenced their recovery progress and partially masked the effects of the EEG intervention. Recent studies have also shown that exercise can improve cognitive impairment in older adults [38]. Future research could further explore the combined effects of EEG intervention and physical activity on rehabilitation outcomes.

Overall, this pilot study provides preliminary evidence that EEG-based intervention may be associated with cognitive improvement in patients with stroke. Although the magnitude of improvement was similar across groups, with no statistically significant differences, the findings should be interpreted cautiously because of the absence of a control group and the potential influence of concurrent rehabilitation therapies. Nevertheless, the results support the feasibility of EEG-based cognitive rehabilitation across different demographic subgroups and warrant further validation in larger randomized controlled studies. This observation is generally consistent with previous studies reporting the feasibility of EEG-based interventions across diverse patient populations [39].

## 5. Conclusions

Our findings suggest possible potential clinical relevance of EEG-based interventions in stroke rehabilitation. The results indicate that EEG interventions may be associated with changes in cognitive outcomes across patients with different demographic characteristics. However, given the single-group pre–post design and the absence of a control group, these findings should be interpreted with caution, and the observed changes cannot be solely attributed to the EEG intervention.

Further well-designed randomized controlled trials with larger sample sizes are warranted to validate these preliminary findings and to determine the effectiveness of EEG interventions in stroke rehabilitation. Future research should also explore optimal intervention protocols and the potential applicability of individualized rehabilitation strategies in different patient populations. However, due to the single-group pre–post design and absence of a control group, causal relationships cannot be established, and the findings should be interpreted as preliminary evidence. Nevertheless, our results provide initial support for the feasibility and clinical potential of EEG-based cognitive training as a complementary rehabilitative approach during acute stroke recovery.

## Figures and Tables

**Table 1:** T-score guidelines for CPT3 ^1^.

T-Score	For Hit Reaction Time (HRT)	T-Score	For All Other Variables
70+	Atypically slow	70+	Very elevated
60–69	Slow	60–69	Elevated
55–59	A little slow	55–59	High Average
45–54	Average	45–54	Average
40–44	A little fast	<45	Low
<40	Atypically fast		

^1^ The guidelines in the following table apply to all T-scores in this report.

**Table 2:** Baseline characteristics of the study population (N = 30).

Variable	Value
Age (years), mean ± SD	70.0± 13.0
Age > 70 years, n (%)	16 (53.3%)
Sex, n (%)	
Male	18 (60.0%)
Female	12 (40.0%)
Stroke type, n (%)	
Ischemic stroke	22 (73.3%)
Hemorrhagic stroke	8 (26.7%)
Time since stroke (<3 months)	30 (100.0%)
Baseline cognitive score (MMSE), mean ± SD	20.83 ± 1.82

**Table 3:** Comparison of EEG-derived attention index scores between the first and tenth intervention sessions (N = 30).

Session	EEG-Derived Attention Index ^1^ (Mean ± SD)	T-Value	*p*-Value
Session 1	46.30 ± 12.11		
Session 10	72.57 ± 11.52	−8.668	0.000

^1^ Data are presented as mean ± standard deviation (SD). The EEG parameter represents the attention index generated by the device algorithm (0–100%). Statistical comparisons were performed using paired *t*-tests between Session 1 and Session 10.

**Table 4:** Pre- and post-test Mini-Mental State Examination (MMSE) scores (sessions 1 and 10) (N = 30).

	MMSE Avg ± SD	T-Value	*p*-Value	R^2^
Session 1	20.83 ± 1.82			
Session 10	24.80 ± 2.75	−10.64	<0.001	0.67

**Table 5:** Pre- and post-test Conners’ Continuous Performance Test (CPT3) scores (sessions 1 and 10) (N = 30).

	CPT Scores Avg ± SD	T-Value	*p*-Value	R^2^
Session 1	71.63 ± 12.61			
Session 10	65.63 ± 15.28	2.906	0.007	0.69

## Data Availability

The data presented in this study are not publicly available due to privacy and ethical restrictions. The dataset contains potentially identifiable information and is subject to institutional regulations and Institutional Review Board (IRB) approval. Relevant raw data supporting the findings have been provided as part of the submission. Additional data are available from the corresponding author upon reasonable request and with permission from the institution.

## Abbreviations

The following abbreviations are used in this manuscript:

EEG	electroencephalogram
MMSE	Mini-Mental State Examination
CPT3	Conners’ Continuous Performance Test
WHO	The World Health Organization
MCI	mild cognitive impairment
TIA	transient ischemic attack
CT	computed tomography
MRI	magnetic resonance imaging
